# Revisiting Post-stimulus Theta Activity: Evidence for a Broadband Rather Than Narrowband Spectral Origin

**DOI:** 10.1523/JNEUROSCI.1194-26.2026

**Published:** 2026-10-09

**Authors:** Theo Vanneau, Michael Quiquempoix, Bradley Voytek, Mate Gyurkovics, Sophie Molholm

**Affiliations:** ^1^The Cognitive Neurophysiology Laboratory, Departments of Pediatrics & Neuroscience, Albert Einstein College of Medicine, Bronx, New York 10461; ^2^Institut de Recherche Biomédicale des Armées (IRBA), Brétigny-sur-Orge 91223, France; ^3^URP 7330 VIFASOM, Université Paris Cité, Hôtel Dieu, Paris 75004, France; ^4^Department of Cognitive Science, UC San Diego, La Jolla, California 92093; ^5^School of Psychology, University of East Anglia, Norwich NR4 7TJ, United Kingdom; ^6^The Frederick J. and Marion A. Schindler Cognitive Neurophysiology Laboratory, The Ernest J. Del Monte Institute for Neuroscience, Department of Neuroscience, University of Rochester School of Medicine and Dentistry, Rochester, New York 14642

**Keywords:** aperiodic activity, brain oscillations, EEG, event-related dynamics, sensory processing, spectral parameterization

## Abstract

The aperiodic, 1/*f*-like component of electrophysiological activity is increasingly recognized as a meaningful feature of neural function rather than background noise. Meanwhile, many EEG studies interpret transient post-stimulus changes in oscillatory power as signatures of attention, salience, or cognitive control. Such interpretations typically depend on baseline normalization, which assumes aperiodic activity remains stable from pre- to post-stimulus periods. Using high-density EEG from typically developing children, we tested this assumption across two paradigms: an audiovisual simple reaction-time task (*n* = 36, 19 males and 17 females) and a visual oddball task (*n* = 38, 20 males and 18 females). In each, conventional spectral analyses were compared with analyses that explicitly modeled and removed aperiodic activity in both windows. Across tasks, stimulus onset produced robust increases in aperiodic exponent and offset, indicating systematic 1/*f* changes. In the audiovisual task these were modality specific, with central, parieto-occipital, or combined topographies by stimulus type. They diminished but persisted after event-related potential removal, suggesting they were not fully explained by phase-locked activity. Critically, once aperiodic activity was accounted for, the apparent theta increase was largely abolished in both tasks, including the canonical fronto-central enhancement to infrequent targets. The conventional method also overestimated beta desynchronization, especially in the induced signal, indicating a spurious consequence of spectral slope steepening. In contrast, alpha desynchronization remained robust and was enhanced, reflecting genuine suppression. Together, these findings suggest many conventional time–frequency effects, particularly apparent theta synchronization, may reflect stimulus-driven aperiodic changes rather than robust narrowband changes, challenging core assumptions of these analyses.

## Significance Statement

Frequency-domain neural activity comprises both narrowband oscillations and broadband aperiodic activity. Conventional time–frequency analyses use baseline normalization to isolate oscillatory responses, implicitly assuming that aperiodic activity remains stable following stimulus onset. Across two EEG paradigms, we show that stimulus presentation systematically alters aperiodic activity in a sensory- and attention-dependent manner. These changes can produce apparent increases in theta-band power that do not reflect genuine narrowband oscillations and can exaggerate beta desynchronization. In contrast, alpha desynchronization remained robust after accounting for aperiodic activity. These findings demonstrate that stimulus-related aperiodic changes can substantially influence conventional time–frequency measures and should be explicitly considered when interpreting post-stimulus oscillatory responses.

## Introduction

Neural activity in the frequency domain reflects contributions from narrowband oscillatory rhythms and broadband aperiodic activity (Pritchard, 1992). Since the earliest EEG studies (Berger, 1929), oscillations have dominated signal interpretation and remain central to basic and clinical neuroscience (Buzsaki and Draguhn, 2004). Neural power spectra, however, are not composed solely of rhythmic peaks. They also contain a broadband component, often termed scale-free or aperiodic activity, that follows a characteristic 1/*f*^x^-like profile in which power decreases as frequency increases (Bullock et al., 1995; Freeman and Zhai, 2009; He et al., 2010; Miller, 2010; He, 2014). EEG, MEG, and local field potentials are therefore better understood as a combination of rhythmic and arrhythmic activity. In event-related recordings, the post-stimulus spectrum additionally contains the contribution of the phase-locked event-related potential (ERP). Because transient ERPs have a broadband spectral representation that can influence both apparent narrowband power and estimated aperiodic parameters, post-stimulus spectra should be examined both before and after ERP removal to distinguish phase-locked evoked activity from non-phase-locked periodic and aperiodic changes (Makeig et al., 2002; Sauseng et al., 2007; Gyurkovics et al., 2022).

This aperiodic component was long treated as background noise and routinely discarded to isolate oscillations (Cohen, 2014; Gyurkovics et al., 2021). Growing evidence now indicates it is itself functionally meaningful and may index ongoing changes in neural state (Manning et al., 2009; Miller et al., 2012; Gao et al., 2017; Donoghue et al., 2020; Waschke et al., 2021; Preston et al., 2025). The aperiodic exponent, in particular, has been linked to excitatory–inhibitory synaptic balance, with steeper spectra thought to reflect relatively greater inhibition (Gao et al., 2017). Its shape may thus mark, moment-to-moment, the dynamics supporting information processing.

Consistent with this, recent work shows the aperiodic component changes following auditory stimulation and that these changes vary with attentional context, such as whether a stimulus is frequent or rare (Gyurkovics et al., 2022). There, post-event spectra differed from pre-event spectra even after removing the time-locked response, largely reflecting a clockwise rotational shift in the 1/*f* component (increased low-frequency, decreased high-frequency power). Its magnitude scaled with attentional demands, suggesting stimulus processing entails systematic aperiodic changes, potentially reflecting increased inhibition after onset.

In classic event-related time–frequency analyses, post-stimulus changes are isolated through baseline correction, which removes pre-stimulus activity from the epoch (Gyurkovics et al., 2021). Within this framework, many studies have reported transient increases in theta-power across diverse tasks and contexts (Cavanagh et al., 2012; Cohen and Donner, 2013; Cavanagh and Frank, 2014; Cavanagh and Shackman, 2015; Gyurkovics and Levita, 2021; McLoughlin et al., 2022; Vanneau et al., 2025b). Frontal or midfrontal theta increases following an event have been linked to control-related processes, with frontal-midline theta often characterized as a marker of control demand, attentional allocation, or performance monitoring rather than any single operation (Cavanagh and Frank, 2014). Theta likewise increases with cognitive load during working memory (Jensen and Tesche, 2002; Hsieh and Ranganath, 2014), yet recent work suggests this increase may be largely artifactual, arising from concurrent changes in aperiodic offset and exponent during retention rather than from genuine periodic theta (Frelih et al., 2025; van Engen et al., 2026).

While theta oscillations are unambiguously prominent in rodent hippocampus (Buzsaki et al., 2003), it remains unclear whether transient theta increases in human cortical recordings reflect true oscillatory activity or are largely driven by aperiodic changes. To address this, we reanalyzed high-density EEG recorded in typically developing children (8–13 years old) across two tasks. The first, an audiovisual simple reaction-time task with stimuli from different sensory modalities, let us test whether aperiodic changes vary systematically with sensory input (Sakowitz et al., 2000; Vicentin et al., 2024; Vanneau et al., 2025a). The second was a classical visual oddball task, in which many studies report enhanced transient fronto-central theta to rare targets relative to standards (Karakas et al., 2000; Bernat et al., 2007; Cavanagh et al., 2012; Harper et al., 2019). By comparing conventional spectral analyses with analyses that explicitly model and remove the aperiodic component, we asked whether these apparent theta effects reflect genuine periodic activity or the aperiodic background.

Throughout this study, “periodic” and “aperiodic” refer to the spectral components identified through parameterization: narrowband peaks above the spectral background and the broadband component, respectively. This distinction does not necessarily map onto separate physiological generators. A smooth 1/*f*-like spectrum may receive contributions from irregular synaptic currents (Gao et al., 2017), transient or burst-like oscillations, ERPs (Gyurkovics et al., 2022), and non-neural sources (Schmidt et al., 2025); therefore, an aperiodic spectral component should not be interpreted as evidence that its underlying activity is exclusively nonoscillatory (Brake et al., 2024; Preston et al., 2026).

## Materials and Methods

### Ethics approval and consent to participate

This study was approved by the Institutional Review Board of the Albert Einstein College of Medicine (IRB # 2021-13433). All participants assented to the procedures and parents/guardians provided informed consent.

### Participants

Data collected for a different project were reanalyzed for the current study. The study initially included 41 typically developing children between 8 and 14 years of age. After excluding participants based on eye-tracking and EEG quality (see respective methods section for more details), the final analysis was conducted on 36 (10.43 years old ± 1.8; mean ± SD, 19 males and 17 females) participants for the audiovisual task and 38 (10.44 years old ± 1.8, 20 males and 18 females) for the visual oddball task. Participants met the following inclusion criteria: no history of neurological, developmental, or psychiatric disorders, no first-degree relatives diagnosed with autism, and enrollment in age-appropriate grade in school. All procedures were approved by the Institutional Review Board of the Albert Einstein College of Medicine and adhered to the ethical standards outlined in the Declaration of Helsinki. All participants assented to the procedures, and their parents or guardian signed an informed consent approved by the Institutional Review Board of the Albert Einstein College of Medicine. Participants received nominal recompense for their participation (at $15 per hour).

### Audiovisual task: experimental procedure (*n* = 36)

Participants were seated in a chair in an electrically shielded room (International Acoustics), 70 cm away from the visual display (Dell UltraSharp 1704FPT). The stimuli, controlled by Presentation software (Neurobehavioral Systems), included three types: a red disk (“Visual”), a 1,000 Hz tone (“Audio”), and their simultaneous presentation (“Audiovisual”). Participants were instructed to press a button as quickly as possible upon detecting any stimulus. The auditory stimulus was 1,000 Hz, 60 ms tone presented binaurally (75 dB SPL). The visual stimulus was a red disk subtending to 1.5°, displayed above a fixation cross. The audiovisual stimulus was a simultaneous presentation of both. Each trial presented a pseudorandomly chosen stimulus (A, V, or AV; represented equiprobably), with stimuli delivered through headphones (HD 650 Sennheiser) and displayed on a flat-panel LCD (Dell UltraSharp 1704FPT, 60 Hz). A jittered randomly sampled interstimulus interval (1,000–3,000 ms) reduced onset predictability and therefore anticipatory responses in the baseline period. The task consisted of 400 trials across four blocks (100 trials per block), each block lasting ∼3 min and 40 s. Button presses were recorded using a response pad (Logitech Wingman Precision Gamepad). Triggers indicating stimulus onset were sent to the EEG stimulus channel from the PC acquisition computer via Presentation software.

### Visual oddball task: experimental procedure (*n* = 38)

Participants were seated in a chair in an electrically shielded room (International Acoustics), 70 cm away from the visual display (Dell UltraSharp 1704FPT). The stimuli, controlled by Presentation software (Neurobehavioral Systems), were objects, each shown as upright and inverted images, along with infrequently presented shadow versions (Fig. 4*A*). The objects come from the BOSS database (Brodeur et al., 2010). Participants were instructed to press a button as quickly as possible upon detecting a shadow stimulus (presented at 20% probability; see Fig. 4*A* for illustration). A jittered interstimulus interval (900–1,100 ms) reduced onset predictability. The task comprised 360 trials across six blocks (60 trials/block, ∼1 min per block).

### EEG recordings and preprocessing

EEG data were recorded at a sampling rate of 512 Hz using 64 channels BioSemi Active II system (using the CMS/DRL referencing system) with an antialiasing filter (−3 dB at 3.6 kHz). Analyses were conducted in Python (3.11) using MNE (Gramfort et al., 2013) and custom scripts available at https://github.com/tvanneau/Ap-corrected. Bad channel detection was performed using the function NoisyChannels (with RANSAC) from the pyprep toolbox (Bigdely-Shamlo et al., 2015). If >15% of the channels were detected as bad, the participant was rejected. Bad channels were interpolated using spline interpolation (Perrin et al., 1989). EEG was filtered using a FIR bandpass filter (0.01–40 Hz), and independent component analysis (ICA) on 1 Hz high-pass EEG was used to identify and manually reject eye-related components (blinks/saccades). For both tasks, epochs were created from −500 to +800 ms around each stimulation with a baseline correction from −50 to +20 ms stimulus onset and referenced to a common average reference.

### Spectral analysis pipeline

The same analysis pipeline was applied to both tasks. For each epoch, we defined a pre-stimulus window from −500 to 0 ms and a post-stimulus window from 0 to 500 ms (Fig. 1*A*). Power spectral density (PSD) was estimated separately within each window using Welch's method with a Hann taper and constant detrending. Spectra were computed on a trial-by-trial basis for each EEG channel in the 2–35 Hz range (Fig. 1*B*) and then averaged across trials within each participant and condition (Fig. 1*C*). Results were robust to the choice of frequency range, as analyses conducted over 3–40 Hz yielded comparable findings. Frequency-band measures were subsequently derived for theta (4–8 Hz), alpha (8–13 Hz), beta (13–25 Hz), and low gamma (25–35 Hz). All analyses were performed on both the total signal and on ERP-removed data. For the latter, the condition-specific evoked response was first computed by averaging trials in the time domain and subtracted from each trial, thereby removing ERPs that have a broadband representation in the frequency domain and could thus have confounded aperiodic estimates (Gyurkovics et al., 2022), and the same PSD pipeline was then applied to these ERP-removed epochs. To compare conventional and aperiodic-aware estimates of stimulus-related spectral change, we implemented two parallel approaches. In the conventional approach, pre- and post-stimulus spectra were log-transformed and spectral change was quantified as the difference between post- and pre-stimulus PSD, i.e., log10(PSD_post_) − log10(PSD_pre_). Band-limited values were then obtained by averaging this spectrum within each frequency band of interest. In the aperiodic-corrected approach, the aperiodic component of both the pre- and post-stimulus spectra was parameterized using the specparam toolbox in fixed aperiodic mode (Fig. 1*D*). Model fitting was restricted to the 2–35 Hz range. For each window, the fitted aperiodic component was subtracted from the log-transformed PSD to obtain an aperiodic-corrected residual spectrum (Fig. 1*E*). Spectral change was then quantified as the difference between the corrected post- and pre-stimulus spectra, i.e., [log10(PSD_post_) − Aperiodic_post_] − [log10(PSD_pre_) − Aperiodic_pre_]. Theta, alpha, beta, and gamma values were extracted from this residual change spectrum in the same way as for the conventional approach. In addition, the aperiodic parameters themselves were retained for both pre- and post-stimulus windows at each channel, including offset, exponent, model goodness-of-fit (*R*^2^), and fitting error.

**Figure 1.**
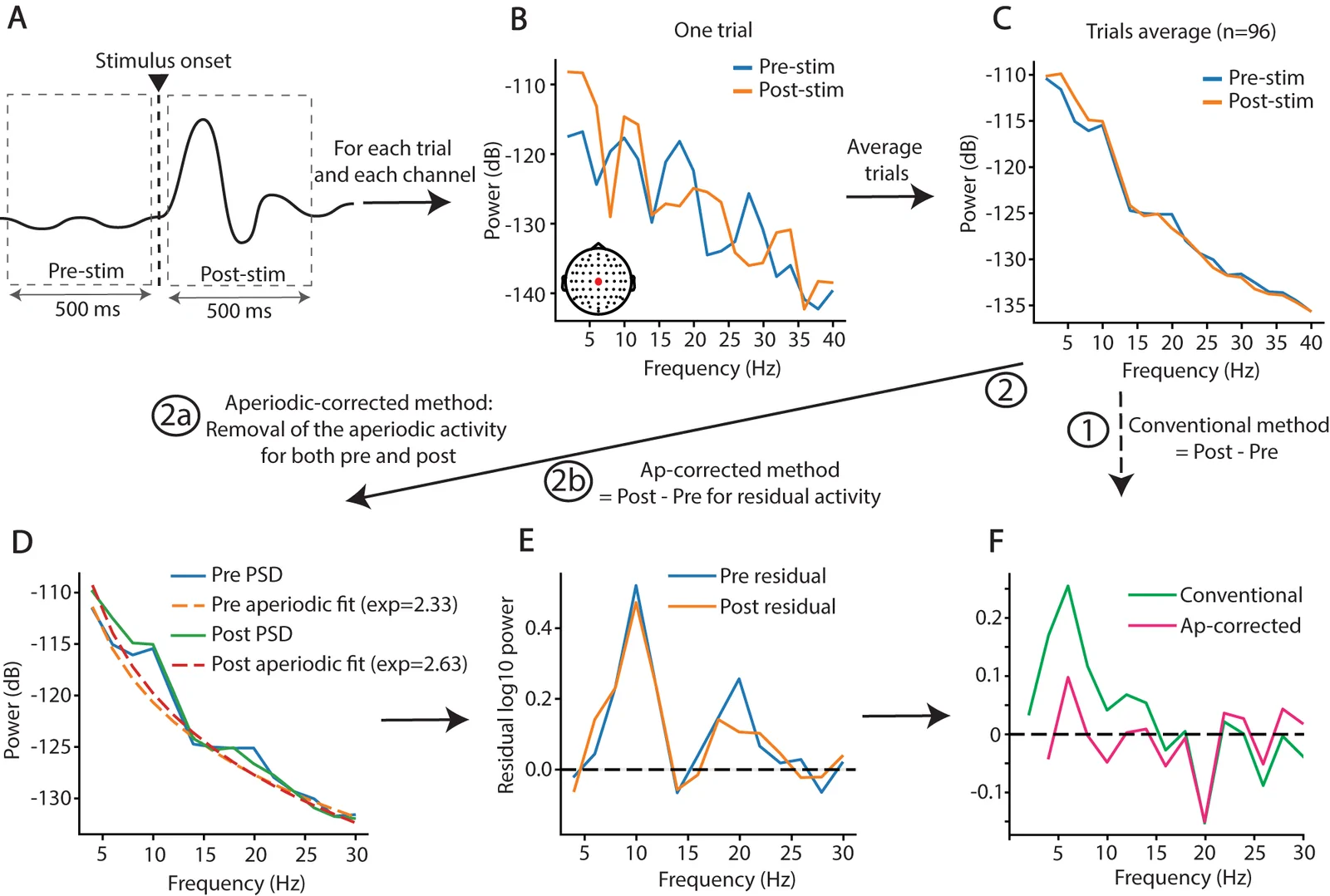
Spectral analysis pipeline. ***A***, For each trial, pre-stimulus (−500 to 0 ms) and post-stimulus (0 to 500 ms) windows were extracted. ***B***, Power spectral density (PSD) was computed separately for each window using Welch's method with a Hann taper and constant detrending. ***C***, Trial-wise PSDs were averaged within each stimulus condition. From these spectra, two complementary approaches were used to quantify stimulus-related spectral change: **(1)**, a conventional approach based on log-transformed pre/post spectral change, and **(2)**, an aperiodic-corrected approach. ***D***, The aperiodic component of the pre- and post-stimulus spectra was parameterized independently using the specparam toolbox (Donoghue et al., 2020). ***E***, Aperiodic-corrected residual spectra were obtained by subtracting the fitted aperiodic component from each window-specific PSD. ***F***, Spectral change was then quantified as the post-minus-pre difference, either from the uncorrected spectra (conventional method, green) or from the aperiodic-corrected residual spectra (aperiodic-corrected method, magenta).

### Statistical analysis

All statistical analyses were conducted using Jamovi (The jamovi project, version 2.3.28; https://www.jamovi.org) for linear mixed-effects models (LMEs) and Python (MNE-Python) for spatial permutation-based testing. Changes in band-limited spectral activity were analyzed using LME in order to account for repeated measurements within individuals. The fixed factors were condition (auditory, visual, audiovisual for the audiovisual task; standard, target for the oddball task) and method (conventional, aperiodic-corrected). Participant was included as a clustering variable with a random intercept, capturing between-participant variability in baseline responses. Fixed effects were evaluated using Type III Wald *F* tests (fixed effects omnibus tests), which test each fixed effect at the parameter estimates of the fitted model while holding all other effects constant. This approach does not involve sequential comparison of nested models; rather, it provides an omnibus test of each effect within the single fitted model, analogous in logic to a conventional ANOVA but conducted within the mixed-effects framework. For each model, we report the omnibus *F* statistic, the associated degrees of freedom, and the corresponding *p* value. As a standardized measure of effect size, we report partial eta squared (*η*^2^p), computed from the *F* statistic and its associated degrees of freedom as *η*^2^p = (*F* × df_effect) / (*F* × df_effect + df_error), which provides an approximation of the proportion of variance in the outcome attributable to each fixed effect after accounting for other terms in the model. Statistical significance was set at *α* = 0.05. Residual normality was evaluated using the Shapiro–Wilk test. To identify brain regions showing significant changes in aperiodic parameters (offset and exponent), we used a nonparametric spatial cluster-based permutation test (Maris and Oostenveld, 2007) as implemented in MNE-Python. For each condition, we first computed subject-level change values at each electrode as the difference between post- and pre-stimulus estimates. These channel-wise change scores were then entered into a one-sample cluster-based permutation test against zero across participants. Clusters were formed over spatially adjacent electrodes using the BioSemi64 sensor adjacency matrix, with a two-sided cluster-forming threshold corresponding to *α* = 0.01. Cluster significance was assessed using 5,000 permutations based on random sign-flipping of the subject-level change values under the null hypothesis of no systematic pre–post difference. For each permutation, the maximum cluster-level statistic was retained to construct the null distribution, and observed clusters were assigned *p* values by comparison with this distribution. In this implementation, the cluster-level statistic corresponded to cluster mass, that is, the sum of *t* values within each spatial cluster (Maris and Oostenveld, 2007).

## Results

### Stimulus-evoked changes in aperiodic activity are modality specific and persist after ERP removal

Examining aperiodic activity before and after stimulus onset revealed consistent findings across both paradigms. In the multisensory task, all sensory conditions were associated with a stimulus-related increase in the aperiodic exponent, indicating a steepening of the 1/*f* component following stimulation. Although this increase was present across conditions, its topographical distribution was modality specific (Fig. 2*B*): auditory stimulation produced a predominantly central pattern, visual stimulation a more parieto-occipital pattern, and audiovisual stimulation a combination of both. Similar results were observed for the aperiodic offset. In the visual oddball task, a significant post-stimulus increase in both the aperiodic exponent and offset was observed for both standard and target trials, with significant clusters spanning all channels for the total signal and a large central region after ERP removal (Fig. 4*B*), indicating a stimulus-induced steepening of the aperiodic slope accompanied by a positive shift in offset regardless of attentional demands.

**Figure 2.**
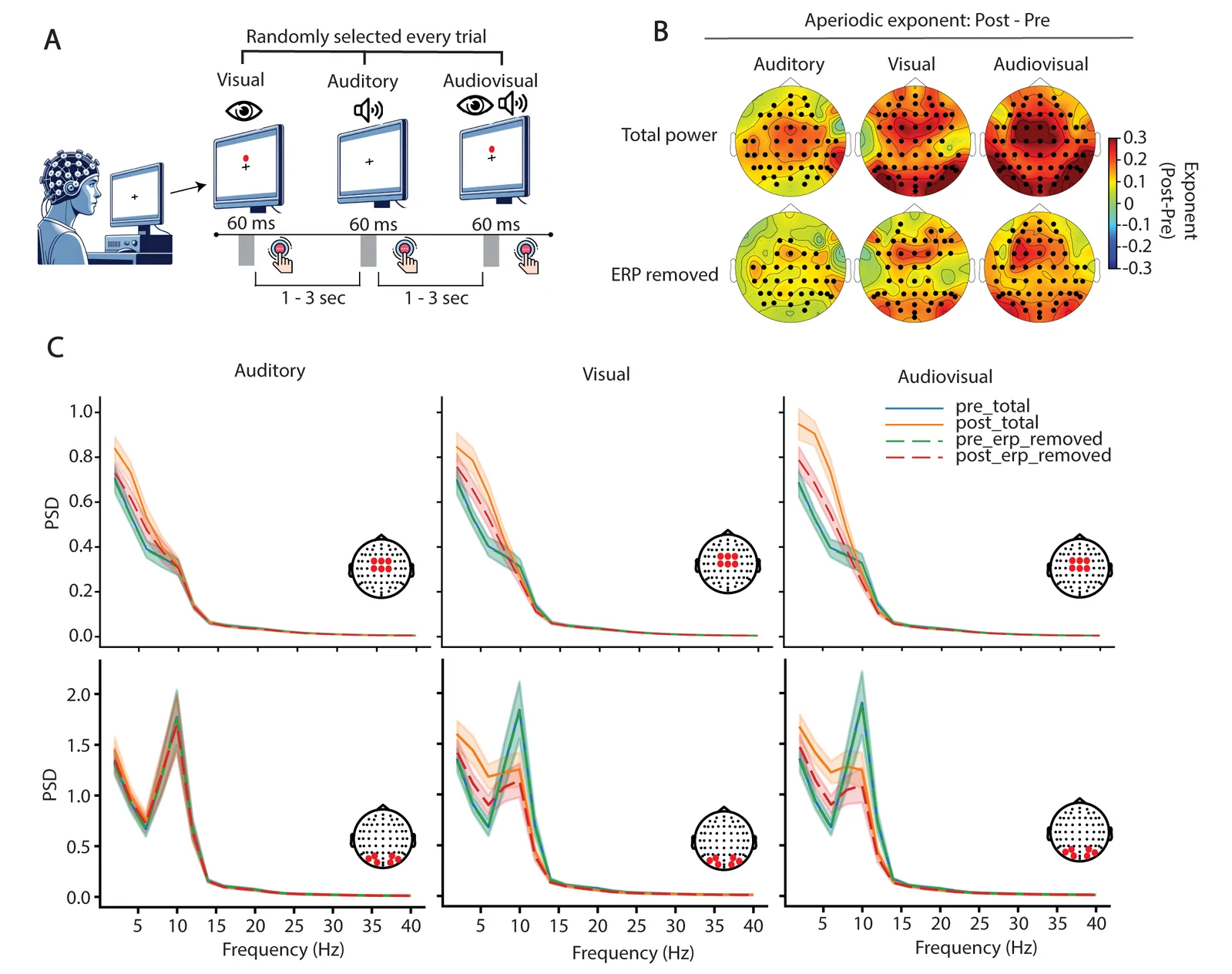
Aperiodic activity changes following stimulus onset and varies as a function of sensory modality. ***A***, Schematic of the audiovisual simple reaction-time task. Visual-only, auditory-only, and audiovisual stimuli were presented in random order, and participants were instructed to respond to every stimulus. ***B***, Topographical maps of the change in aperiodic exponent from pre- to post-stimulus window (post − pre) for each stimulus condition. Results are shown for the total signal (top row) and after time-domain removal of the ERP (bottom row). Black dots indicate channels belonging to significant spatial clusters identified using cluster-based permutation testing against zero change. ***C***, Power spectral density (PSD, linear domain; see Supplementary Fig. 1 for PSD in log) in the pre-stimulus window (blue) and post-stimulus window (orange), shown for the total signal (solid lines) and after time-domain ERP removal (dashed lines), for auditory (left), visual (middle), and audiovisual (right) stimulation. Spectra are averaged across a fronto-central channel cluster (top row) and a parieto-occipital channel cluster (bottom row).

Across both paradigms, time-domain ERP removal reduced the magnitude of these aperiodic effects but did not eliminate them: significant spatial clusters remained and, in the multisensory task, preserved the same overall modality-specific topographical organization (Fig. 2*B*). This was further supported by PSDs extracted from central and parieto-occipital channel clusters for each sensory condition, where post-stimulus spectra showed clear changes in offset and exponent relative to the pre-stimulus window that remained visible after ERP removal, albeit attenuated (Fig. 2*C*). Together, these findings indicate that the ERP contributes to stimulus-related aperiodic changes but does not fully account for them. Importantly, these broadband spectral shifts reshaped the spectral profile in ways that generate an apparent increase in theta-range power and reduce the observed magnitude of alpha desynchronization.

### Theta-band: aperiodic correction abolishes the apparent stimulus-related increase

Prior to aperiodic correction, theta-band power changes displayed a topographical pattern closely mirroring aperiodic effects. In the multisensory task, auditory stimulation was associated with increased theta-band power over central channels, visual stimulation with increases over both central and parieto-occipital channels, and audiovisual stimulation with a similar but spatially broader pattern (Fig. 3*A*). Following aperiodic correction, the theta-band increase was greatly reduced for all stimulus types. In the visual oddball task, a similar pattern was observed, with fronto-central theta increases for both standard and target stimuli that were substantially reduced after aperiodic correction (Fig. 4*A*).

**Figure 3.**
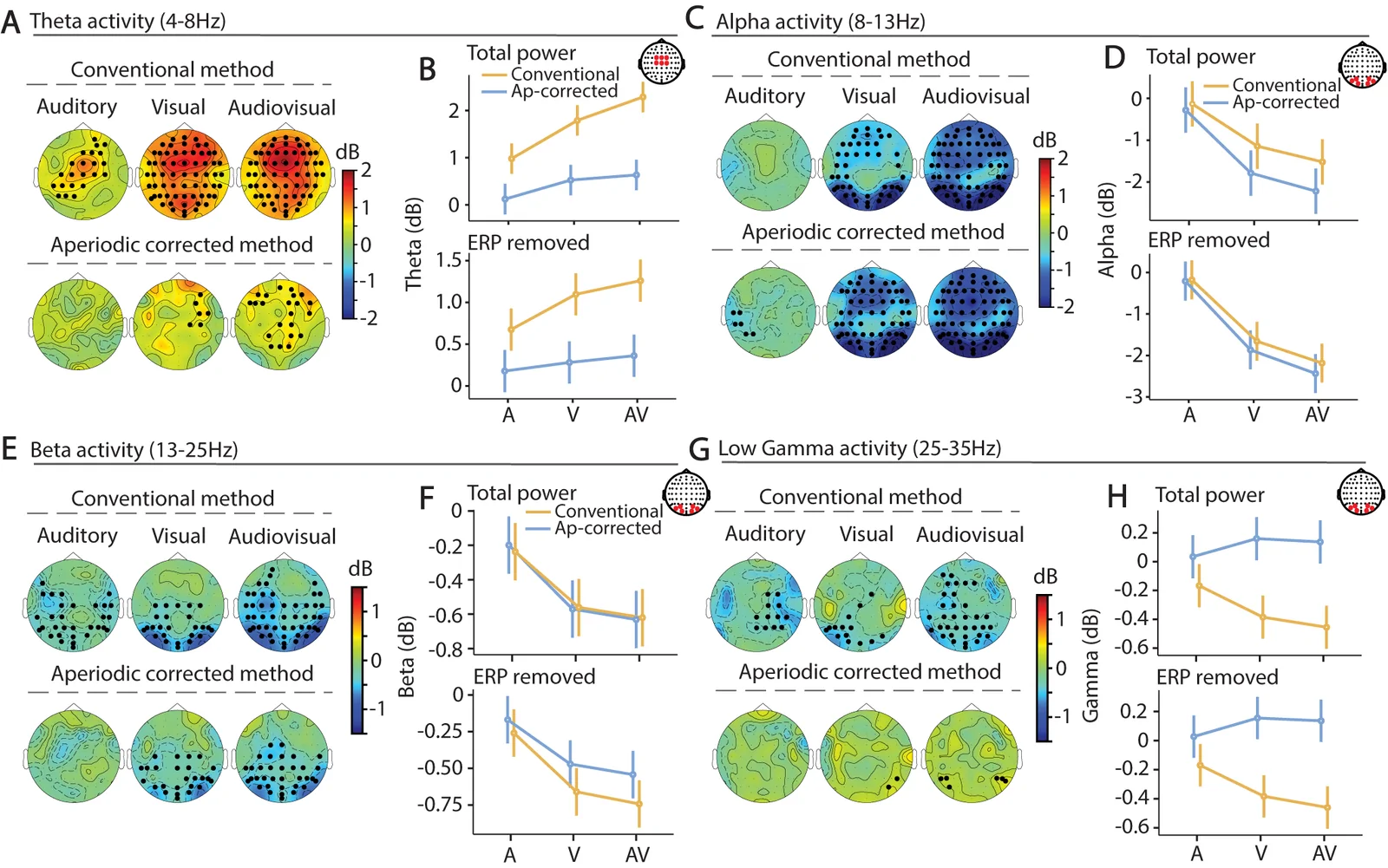
Aperiodic shifts explain apparent theta and gamma changes but not alpha and beta desynchronization across sensory modalities. ***A***, Topographical representation of theta-band (4–8 Hz) changes in response to auditory (left), visual (middle), and audiovisual (right) stimulation after removal of the ERP. Theta was quantified either with the conventional approach (top row; post − pre change in log-transformed PSD) or after aperiodic correction of both pre- and post-stimulus spectra (bottom row; see Materials and Methods). ***B***, Theta-band values averaged across a fronto-central channel cluster for each stimulus condition, shown for the conventional method (orange) and the aperiodic-corrected method (blue), for the total signal (top panel), and ERP-removed signal (bottom panel). ***C***, ***D***, Same as ***A*** and ***B*** for alpha-band activity (8–13 Hz); ***E***, ***F***, for beta-band activity (13–25 Hz); ***G***, ***H***, for low gamma-band activity (25–35 Hz).

**Figure 4.**
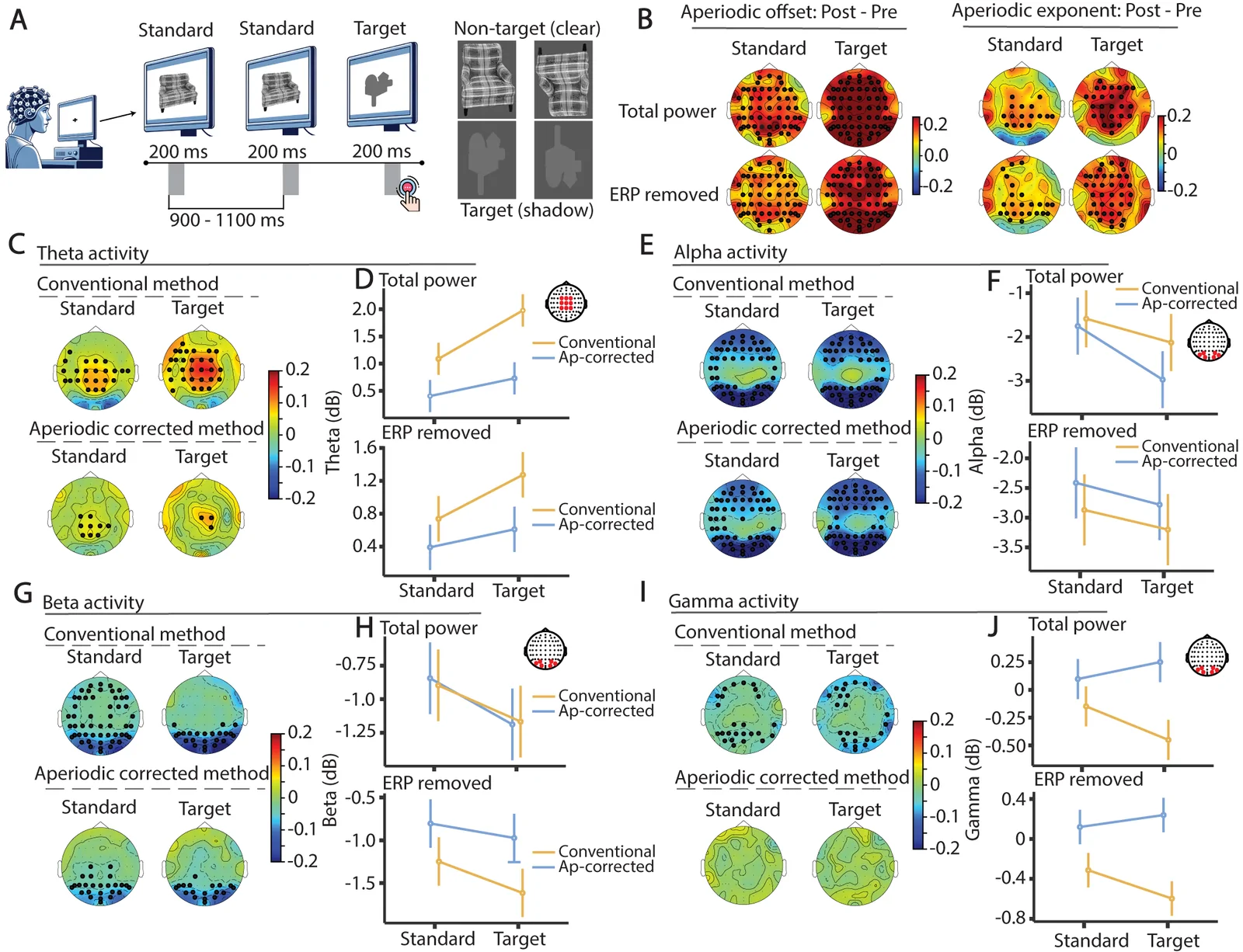
Aperiodic activity accounts for most stimulus-related theta changes, but not alpha changes, during the visual oddball task. ***A***, Schematic of the visual oddball task. Participants were presented with upright and inverted clear object stimuli (standards) and shadow versions (targets) and were instructed to respond only to target stimuli. ***B***, Topographical maps of the change in aperiodic exponent from pre- to post-stimulus window (post − pre) for standard and target stimuli, shown for the total signal (left) and after time-domain ERP removal (right). Black dots indicate channels belonging to significant spatial clusters identified with cluster-based permutation testing against zero change. ***C***, Topographical maps of theta-band (4–8 Hz) changes in response to standard (left) and target (right) stimuli, computed either with the conventional method (top row; post − pre change in log-transformed PSD) or after aperiodic correction of both pre- and post-stimulus spectra (bottom row; see Materials and Methods). ***D***, Mean theta-band values averaged across a fronto-central channel cluster for standard and target stimuli, shown for the conventional method (orange) and the aperiodic-corrected method (blue), for the total signal (top panel) and ERP-removed signal (bottom panel). ***E***, ***F***, Same as ***C*** and ***D*** for alpha-band activity (8–13 Hz); ***G***, ***H***, for beta-band activity (13–25 Hz); and ***I***, ***J***, for low gamma-band activity (25–35 Hz).

To formally test whether each method yielded statistically significant pre- to post-stimulus changes in theta-band power, we conducted spatial cluster-based permutation tests separately for each method and each sensory condition. In the multisensory task, the conventional method yielded a significant spatial cluster confined to central channels for auditory stimulation, and clusters encompassing a much broader scalp distribution for both visual and audiovisual stimulation. After aperiodic correction, no significant theta-band cluster was found for auditory stimulation, and only small, spatially restricted clusters emerged for visual (right frontal) and audiovisual (frontal and parietal) stimulation, indicating that the pre- to post-stimulus theta-band change is substantially reduced and largely nonsignificant once aperiodic activity is accounted for (Fig. 3*A*). In the oddball task, both standard and target stimuli yielded significant fronto-central theta clusters for the conventional method; after aperiodic correction, no significant clusters were observed for either condition (Fig. 4*A*).

Given that the largest theta-band changes were consistently observed over fronto-central channels, and that this region showed maximal effect amplitudes across conditions, theta-band power changes were averaged across a fronto-central ROI (Cz, FC1, FCz, FC2, Fz) and submitted to LMEs with stimulus condition and method (conventional vs aperiodic-corrected) as fixed factors and subject as a random factor. Models were conducted separately for the total signal and the ERP-removed signal (see Supplementary Fig. 2 for boxplots).

In the multisensory task and for the total signal, there was a significant main effect of condition (*F*_(2,175)_ = 36.73, *η*^2^p = 0.30, *p* < 0.001), with theta-band changes increasing progressively from auditory to visual to audiovisual stimulation, and significant pairwise differences between all three conditions (all *p*_s_ ≤ 0.017). There was also a robust main effect of method (*F*_(1,175)_ = 203.35, *η*^2^p = 0.54, *p* < 0.001), with substantially smaller theta-band changes after aperiodic correction (Δ = 1.28 dB ± 0.08, *t* = 14.3). Critically, the condition × method interaction was significant (*F*_(2,175)_ = 6.76, *η*^2^p = 0.07, *p* < 0.001): whereas the conventional method showed clear differences across all stimulus conditions, these differences were markedly reduced after aperiodic correction, with only the auditory versus audiovisual comparison remaining significant and with a much smaller effect size (conventional: *t* = 8.55, *p* < 0.001; aperiodic-corrected: *t* = 3.35, *p* = 0.015; V vs AV for conventional: *t* = 3.25, *p* = 0.02; aperiodic-corrected: *t* = 0.7, *p* = 1.0). The same general pattern was observed after ERP removal, with theta-band changes remaining significantly larger for the conventional method (*F*_(1,175)_ = 96.31, *η*^2^p = 0.35, *p* < 0.001), though the condition × method interaction did not reach significance (*F*_(2,175)_ = 2.66, *η*^2^p = 0.03, *p* = 0.072).

A strikingly similar pattern emerged in the visual oddball task. For the total signal, there was a significant main effect of condition (*F*_(1,111)_ = 24.43, *η*^2^p = 0.18, *p* < 0.001), with greater theta-band increases for targets than for standards (Δ = 0.6 dB ± 0.1, *t* = 4.94, *p* < 0.001), and a significant main effect of method (*F*_(1,111)_ = 61.71, *η*^2^p = 0.36, *p* < 0.001), reflecting larger theta-band changes in the conventional analysis (Δ = 1.0 dB ± 0.1, *t* = 7.86, *p* < 0.001). The condition × method interaction was significant (*F*_(1,111)_ = 5.27, *η*^2^p = 0.05, *p* = 0.02): the target-related theta enhancement was present with the conventional method (Δ = 0.9 dB ± 0.1, *t* = 5.12, *p* < 0.001) but was no longer significant after aperiodic correction (Δ = 0.3 dB ± 0.1, *t* = 1.87, *p* = 0.38). After ERP removal, the main effects of condition and method remained significant (*p*s < 0.001), and although the interaction did not reach significance (*F*_(1,111)_ = 2.03, *η*^2^p = 0.01, *p* = 0.15), the same overall pattern was observed: target-related theta enhancement remained significant for the conventional method (*p* = 0.005) but not for the aperiodic-corrected method (*p* = 0.99).

As an exploratory analysis, we contrasted theta-band changes before and after ERP removal separately for each method and task. In both paradigms, ERP removal was associated with a significant reduction in theta-band change magnitude for the conventional method (multisensory: *F*_(1,175)_ = 74.34, *η*^2^p = 0.3, *p* < 0.001; oddball: *F*_(1,111)_ = 20.31, *p* < 0.001), but this effect was absent or negligible for the aperiodic-corrected method (multisensory: *F*_(1,175)_ = 3.93, *η*^2^p = 0.02, *p* = 0.049; oddball: *F*_(1,111)_ < 0.1, *p* = 0.93). This indicates that the conventional method is considerably more sensitive to ERP contamination than the aperiodic-corrected method and that a large proportion of the theta-band change it detects reflects the ERP rather than oscillatory dynamics.

Together, and across both paradigms, these results indicate that the apparent stimulus-related theta-band increase is largely explained by concurrent changes in aperiodic activity rather than by a genuine increase in oscillatory theta-power.

### Alpha-band: effects remain present even after aperiodic correction

Unlike theta, alpha-band activity was characterized by a decrease in power following stimulus onset, consistent with event-related alpha desynchronization. In the multisensory task, auditory stimulation produced only modest desynchronization primarily over temporal channels, whereas visual and audiovisual stimulation elicited a much stronger desynchronization over parieto-occipital regions (Fig. 3*C*). In the oddball task, the spatial distribution was very similar between standard and target stimuli and between methods, with significant parieto-occipital and frontal clusters identified for all conditions (Fig. 4*E*). Notably, in both paradigms, aperiodic correction modulated the magnitude of alpha desynchronization, consistent with the idea that stimulus-related changes in aperiodic activity partially mask the true extent of alpha suppression.

At the cluster level in the multisensory task, significant alpha-band parieto-occipital clusters were identified by both methods for visual and audiovisual stimulation, but the aperiodic-corrected method additionally revealed a significant temporal cluster absent from the conventional analysis and produced a spatially broader central cluster for visual stimulation, a pattern that stands in clear contrast to the theta-band findings. In the oddball task, significant clusters were identified by both methods for standard and target stimulation across all conditions.

Alpha changes were averaged across parieto-occipital ROI (O1, Oz, O2, PO3, PO7, PO4, and PO8) and submitted to LMEs. In the multisensory task and for the total signal, there was a significant main effect of condition (*F*_(2,175)_ = 49.42, *η*^2^p = 0.36, *p* < 0.001), with stronger alpha desynchronization for visual and audiovisual stimuli than for auditory stimuli (A vs V: Δ = 1.25 dB, *t* = 7.22, *p* < 0.001; A vs AV: Δ = 1.66 dB, *t* = 9.53, *p* < 0.001), and a significant main effect of method (*F*_(1,175)_ = 12.17, *η*^2^p = 0.07, *p* < 0.001), reflecting greater desynchronization after aperiodic correction (Δ = 0.5 dB, *t* = 3.49, *p* < 0.001), but no significant condition × method interaction (*F*_(2,175)_ = 1.51, *η*^2^p = 0.02, *p* = 0.22). In the oddball task, there was similarly a significant main effect of condition (*F*_(1,111)_ = 40.04, *η*^2^p = 0.26, *p* = 0.005), with stronger alpha desynchronization for target than standard stimuli, a significant main effect of method (*F*_(1,111)_ = 13.18, *η*^2^p = 0.1, *p* < 0.001), and a significant condition × method interaction (*F*_(1,111)_ = 5.91, *η*^2^p = 0.05, *p* = 0.01), with a larger standard-target difference for the aperiodic-corrected method (Δ = 1.2 dB, *t* = 6.19, *p* < 0.001) than for the conventional method (Δ = 0.5 dB, *t* = 2.75, *p* = 0.04).

After ERP removal, the pattern diverged somewhat between paradigms. In the multisensory task, the condition effect remained significant (*F*_(2,175)_ = 102.34, *η*^2^p = 0.54, *p* < 0.001), while neither the main effect of method nor the condition × method interaction reached significance (*p*s > 0.12). In the oddball task, the condition effect again remained significant (*F*_(1,111)_ = 8.31, *η*^2^p = 0.07, *p* = 0.005), but in contrast to the multisensory task, a significant main effect of method emerged with smaller alpha desynchronization for the aperiodic-corrected method (*F*_(1,111)_ = 13.15, *η*^2^p = 0.1, *p* < 0.001), with no significant interaction.

Contrasting alpha changes before and after ERP removal revealed a consistent pattern across paradigms: ERP removal was associated with a significant increase in alpha desynchronization for the conventional method (multisensory: *F*_(1,175)_ = 8.11, *p* = 0.005; oddball: *F*_(1,111)_ = 96.52, *p* < 0.001), indicating that the ERP attenuates the underlying alpha desynchronization signal with this approach. No such effect was observed for the aperiodic-corrected method in either paradigm (multisensory: *F*_(1,175)_ = 0.37, *p* = 0.54; oddball: *F*_(1,111)_ = 2.4, *p* = 0.12).

Together, these results suggest that alpha desynchronization is not primarily driven by changes in aperiodic activity and remains robust after aperiodic correction, in clear contrast to the theta-band findings. Aperiodic correction modulates the estimated magnitude of alpha suppression, likely by removing the attenuating influence of concurrent broadband shifts but does not abolish the effect.

### Beta-band: aperiodic correction reduces the apparent magnitude of desynchronization

Beta-band activity exhibited a post-stimulus decrease in power relative to baseline in both paradigms (Figs. 3*E*, 4*E*). In the multisensory task, significant parieto-occipital beta-band clusters were identified by both methods for visual and audiovisual stimulation, though these were spatially broader for the conventional method, and no significant cluster was observed for auditory stimulation after aperiodic correction. A similar pattern was observed in the oddball task, where both methods yielded significant parieto-occipital clusters for standard and target stimulation, again with broader spatial extent for the conventional method.

Beta-band power was averaged across the parieto-occipital ROI used for alpha and submitted to LMEs. When considering the total signal, results were comparable across both paradigms: only the main effect of condition reached significance (multisensory: *F*_(2,175)_ = 21.68, *η*^2^p = 0.20, *p* < 0.001; oddball: *F*_(1,111)_ = 13.89, *η*^2^p = 0.11, *p* < 0.001), with no significant effect of method or condition × method interaction (all *p*s > 0.6). After ERP removal, the condition effect remained significant in both paradigms (multisensory: *F*_(2,175)_ = 26.16, *η*^2^p = 0.23, *p* < 0.001; oddball: *F*_(1,111)_ = 14.79, *η*^2^p = 0.12, *p* < 0.001), and a significant main effect of method emerged in both, indicating that the conventional method overestimated the magnitude of beta desynchronization relative to the aperiodic-corrected method (multisensory: Δ = 0.16 dB, *t* = 3.1, *p* = 0.002; oddball: Δ = 0.5 dB, *t* = 7.8, *p* < 0.001). The condition × method interaction remained nonsignificant in both paradigms.

Contrasting beta changes before and after ERP removal revealed different patterns for the two methods. For the conventional method, ERP removal was associated with a significant change in beta desynchronization magnitude in the oddball task (*F*_(1,111)_ = 51.57, *p* < 0.001) but not in the multisensory task (*F*_(1,175)_ = 2.86, *p* = 0.09). The aperiodic-corrected method showed no significant effect of ERP removal in either paradigm (multisensory: *F*_(1,175)_ = 2.51, *p* = 0.11; oddball: *F*_(1,111)_ = 3.61, *p* = 0.06), suggesting that ERP contamination does not primarily account for the discrepancy between methods in this frequency band.

Together, these results indicate that while the two methods yield comparable estimates of beta desynchronization for the total signal, the conventional method systematically overestimates the magnitude of beta desynchronization in the ERP-removed signal, consistent with a contribution of aperiodic slope changes to broadband power that disproportionately affects the conventional approach.

### Gamma-band: aperiodic correction reverses the apparent direction of activity changes

Finally, we examined stimulus-related changes in the low gamma-band (25–35 Hz), where the two approaches yielded qualitatively opposite patterns in both paradigms. In the multisensory task, the conventional method identified significant gamma desynchronization clusters across all three conditions, whereas after aperiodic correction no desynchronization was observed; instead, significant gamma-band synchronization emerged, with a right temporal cluster for visual stimulation and bilateral temporal clusters for audiovisual stimulation (Fig. 3*G*). In the visual oddball task, the conventional method similarly identified significant gamma desynchronization clusters across parieto-occipital and frontal channels for both standard and target stimulation, while after aperiodic correction no significant changes in gamma activity were detected (Fig. 4*I*).

LMEs confirmed a highly significant effect of method in both paradigms (multisensory: *F*_(1,175)_ = 58.74, *η*^2^p = 0.25, *p* < 0.001; oddball: *F*_(1,111)_ = 32.72, *η*^2^p = 0.22, *p* < 0.001): while the conventional method indicated desynchronization, the aperiodic-corrected method indicated either synchronization (multisensory) or no change (oddball), with a consistent difference between methods of ∼0.45–0.47 dB. The main effect of condition was not significant in either paradigm, but significant condition × method interactions were observed in both (multisensory: *F*_(2,175)_ = 4.47, *η*^2^p = 0.05, *p* = 0.01; oddball: *F*_(1,111)_ = 7.53, *η*^2^p = 0.06, *p* = 0.007), though no individual post hoc comparisons reached significance in either case.

In contrast to the theta and alpha bands, contrasting gamma changes before and after ERP removal revealed no significant effect of signal type for either method in either paradigm (all *p*s > 0.09), indicating that ERP contamination does not account for the discrepancy between methods in this frequency band.

Taken together, these findings demonstrate that the apparent gamma-band desynchronization detected by the conventional method is a spurious consequence of concurrent changes in aperiodic activity. Specifically, the post-stimulus increases in both the exponent and offset of the aperiodic slope steepens the spectral gradient, artificially suppressing power at higher frequencies and producing the illusion of gamma desynchronization in the absence of any true change in narrowband gamma oscillatory activity.

### Pre-stimulus aperiodic activity is associated with reaction time

We next examined whether aperiodic activity was related to behavioral performance. For the audiovisual task, LMEs were fitted with reaction time as the dependent variable, sensory modality as a fixed factor, and the aperiodic parameter of interest entered as a covariate. These analyses revealed that both pre-stimulus aperiodic parameters were significantly associated with reaction time (Fig. 5*A*), for both the exponent (*F*_(1,102)_ = 4.27, *η*^2^p = 0.04, *p* = 0.041, *β* = 93.7 ms) and the offset (*F*_(1,99)_ = 8.67, *η*^2^p = 0.08, *p* = 0.004, *β* = 100.8 ms). This is consistent with recent evidence suggesting that flatter pre-stimulus aperiodic slopes, reflecting broader cortical excitability, are associated with better perceptual performance (Cunningham et al., 2023). In contrast, post-stimulus aperiodic parameters were not significantly associated with reaction time, possibly reflecting increased spectral variability during the post-stimulus period that reduces the reliability of the aperiodic estimate as a behavioral predictor (Fig. 5*B*). In contrast, post-stimulus aperiodic parameters were not significantly associated with reaction time. For the visual oddball task, reaction times were available only for target stimuli. Because the number of target trials was lower than in the audiovisual task and only a single condition was considered, these results should be interpreted more cautiously. Nonetheless, a similar pattern was observed, with a significant association between pre-stimulus offset and reaction time (Supplementary Fig. 5). Taken together these results suggest that flatter aperiodic activity before the stimulus is associated with faster reaction time.

**Figure 5.**
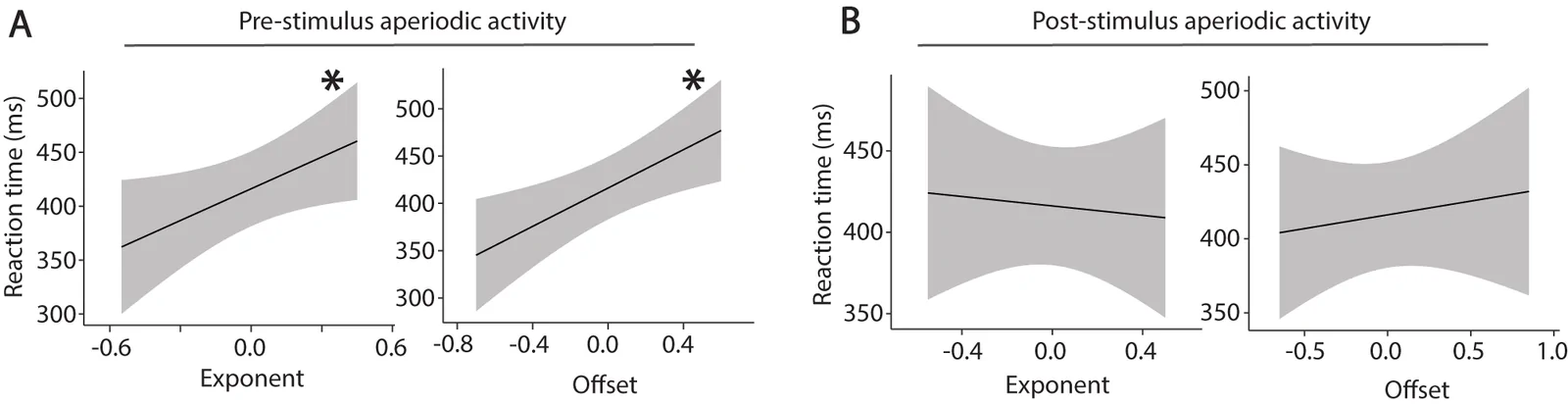
Relationship between aperiodic activity and reaction time. ***A***, Linear mixed-effects model predictions showing the association between pre-stimulus aperiodic exponent (left) and offset (right) and reaction time, while controlling for sensory modality (condition as a fixed effect) and including Subject as a random intercept. Aperiodic parameters are mean centered such that zero corresponds to the sample mean. ***B***, Same analysis for post-stimulus averaged aperiodic parameters.

## Discussion

The present study examined event-related changes in the aperiodic, 1/*f*-like component of the EEG spectrum and their consequences for conventional baseline-normalized analyses. Across two paradigms, stimulus onset reliably steepened the aperiodic exponent, with modality- and task-specific distributions that persisted after ERP removal. Because this change took the form of a clockwise rotation of the power spectrum, it systematically distorted frequency-specific estimates of post-stimulus power: at lower frequencies the rotation inflated power, producing spurious theta increases and partially masking alpha desynchronization, while at higher frequencies it suppressed power, amplifying apparent beta and gamma desynchronization. Conventional analyses thus mischaracterized the direction or magnitude of stimulus-related changes in every band examined. These results suggest many prior reports of post-stimulus theta increases may have overestimated genuine oscillatory contributions and call for caution when interpreting post-stimulus band power with methods that assume aperiodic stationarity.

### Abolishment of apparent theta-power increases after periodic–aperiodic decomposition

This study adds to a growing literature on the limitations of baseline correction, in which pre-stimulus activity is removed from the full epoch of interest (Cohen, 2014; Gyurkovics et al., 2021). Decomposing the signal into periodic and aperiodic components reveals that low-frequency theta largely “disappears” from the periodic component once the aperiodic background is accounted for. This is consistent with recent studies of aperiodic change in working memory and cognitive control (Lendner et al., 2023; Akbarian et al., 2024; Kalamala et al., 2024; Frelih et al., 2025; Preston et al., 2025; Virtue-Griffiths et al., 2025; van Engen et al., 2026), which converge on the conclusion that most stimulus- or task-related theta-power changes in human EEG primarily reflect broadband aperiodic modulation rather than a separable narrowband theta component. We extend these findings by showing aperiodic changes are modality specific, localizing to sensory areas by input modality, consistent with an audiovisual detection task (Waschke et al., 2021), and that attentional demands further modulate aperiodic activity in a visual oddball, paralleling auditory oddball findings (Gyurkovics et al., 2022).

### Reduction of the apparent beta and gamma desynchronization after aperiodic correction

The same logic extends to the higher frequency bands examined here. In the beta-band, the conventional method (subtraction of the full pre-stimulus spectrum from the full post-stimulus spectrum) systematically overestimated the magnitude of desynchronization, particularly in the ERP-removed signal, consistent with the aperiodic slope change suppressing power broadly at frequencies above the spectral rotation point. In the gamma-band, the distortion was most striking: what appeared as gamma desynchronization under conventional analysis was entirely reversed after aperiodic correction, revealing either synchronization or no change, indicating that the apparent gamma suppression was a purely spurious consequence of spectral slope steepening. It is worth noting that the stimuli used in the present study would not be expected to elicit robust gamma increases of the kind typically reported in response to luminance gratings (Hermes et al., 2015). This finding should be interpreted with some caution, however, as gamma-band activity is inherently more difficult to measure reliably with scalp EEG due to its low signal-to-noise ratio and susceptibility to muscular artifacts (Nunez and Srinivasan, 2010; Muthukumaraswamy, 2013). Nonetheless, the consistency of this reversal across both paradigms suggests that in the case the apparent modulation of gamma activity reflects a genuine methodological artifact rather than random noise.

### Alpha desynchronization persists after aperiodic correction

In contrast to theta, beta, and gamma, alpha desynchronization was not diminished by aperiodic correction but enhanced. Corrected alpha suppression exceeded the conventional estimate and remained robust across both paradigms and both total and ERP-removed signals. This is consistent with the slope change partially masking true alpha desynchronization: the broadband upward shift from the rotation attenuates the apparent dip in the alpha range, so correcting for it unmasks stronger suppression. Correction also improved spatial sensitivity, revealing additional temporal clusters in the multisensory task that conventional analysis missed.

Although the oscillatory character of the signal was not measured directly in the time domain (Myrov et al., 2024; Karvat et al., 2026), the increase in alpha effect size after correction suggests that the alpha decrease, unlike the apparent theta increase, reflects suppression of a separable narrowband alpha component rather than being explained by the fitted aperiodic component. This distinction matters functionally. Alpha desynchronization is widely described as an index of cortical excitability (Klimesch et al., 2007): alpha power over sensory cortex decreases with auditory and visual input and is associated with greater excitability, faster reaction times, and more efficient processing (Sauseng et al., 2005; 2009; Romei et al., 2008; Lange et al., 2013; Samaha et al., 2017; Darrell et al., 2026). That correction preserves and amplifies this effect reinforces its status as a reliable index of sensory cortical dynamics, while showing conventional analyses underestimate its magnitude.

The coexistence of an increased aperiodic exponent (suggesting greater inhibitory drive under the E/I balance interpretation; Gao et al., 2017) and alpha desynchronization (suggesting greater excitability) appears contradictory. One resolution is that they operate at different spatial scales: aperiodic changes may reflect a broad, nonfocal inhibitory “braking” signal, while alpha desynchronization reflects a spatially targeted excitability increase over task-relevant regions, potentially with alpha synchronization over task-irrelevant areas, broadly compatible with Gyurkovics et al. (2022). Notably, intracranial recordings during working memory report a decreased exponent concurrent with alpha desynchronization, suggesting both index local excitability (Preston et al., 2025); this divergence may reflect scale-dependent differences in what the aperiodic component indexes.

### The importance of accounting for ERPs in spectral and aperiodic estimation

ERP also contributes to the post-stimulus spectrum. Because ERPs have a broadband spectral representation (Makeig et al., 2002; Min et al., 2007; Sauseng et al., 2007; Gyurkovics et al., 2022), failing to account for them risks attributing ERP-driven power changes to oscillatory or aperiodic mechanisms (Supplementary Figs. 3, 4). Here, ERP removal reduced theta-band changes, indicating that ERPs are an additional, independent source of conflation beyond aperiodic activity. ERP removal also reduced the observed aperiodic changes, showing that accurate quantification of non-phase-locked aperiodic spectral changes likewise requires prior ERP removal (Gyurkovics et al., 2022). Whether the activity underlying the ERP is best characterized as additive and broadband, as a phase reset of ongoing oscillations, or some combination, remains debated and beyond the present scope.

### Functional significance of event-related aperiodic changes

The aperiodic changes we observed overlap spatially and temporally with effects previously attributed to theta, raising the question of whether functions classically linked to stimulus-related theta, including attentional orienting, sensory gating, cognitive control, and working memory updating (Cavanagh et al., 2012; Cohen and Donner, 2013; Cavanagh and Frank, 2014; Cavanagh and Shackman, 2015; Gyurkovics and Levita, 2021; McLoughlin et al., 2022), might be carried, at least partly, by aperiodic activity.

This possibility is reinforced by evidence linking aperiodic activity to diverse cognitive functions. Resting-state aperiodic exponent tracks individual differences in processing speed (Ouyang et al., 2020) and appears to dominate what is traditionally read as “oscillatory” functional connectivity in scalp EEG (Monchy et al., 2025). Pre-stimulus aperiodic activity predicts detection of near-threshold visual stimuli (Cunningham et al., 2023), and post-stimulus steepening tracks attentional filtering in a cued flanker task, with larger steepening associated with smaller flanker interference (Kalamala et al., 2024). Event-related aperiodic changes also track memory load (van Engen et al., 2026); the present data add that pre-stimulus aperiodic exponent predicts individual reaction times in typically developing children, suggesting the aperiodic background at stimulus onset shapes subsequent processing speed.

Taken with the finding that most stimulus-related theta changes reflect aperiodic modulation, this invites a reconsideration of cognition's functional architecture. If correlates of functions previously attributed to oscillatory theta are largely aperiodic, many core operations may reflect coordinated periodic and aperiodic dynamics rather than oscillations alone. Under the E/I interpretation (Gao et al., 2017), transient, spatially specific shifts in local excitation–inhibition balance may be a computational mechanism in their own right rather than a backdrop, with significance depending on which network is affected and on timing, which could explain the aperiodic exponent's link to such heterogeneous functions.

A further consideration is the rotational point of the aperiodic spectrum: the frequency around which the slope pivots as offset and exponent change (Podvalny et al., 2015). Its location determines which bands appear to increase or decrease under the same aperiodic shift, with direct implications for interpreting band-limited power (Gyurkovics et al., 2022; Preston et al., 2025). Here the pivot appears to fall just above the alpha-band, near 13 Hz: theta and alpha power (4–13 Hz) increased in the uncorrected spectrum while beta and gamma (13–40 Hz) decreased, exactly as expected from a steepening slope pivoting at that frequency. Whether this pivot is merely a mathematical artifact of how slope and offset covary or reflects something intrinsic to the circuit remains unclear.

A further limitation is that the study was restricted to children aged 8–13 years, limiting the generalizability of the findings across development. Age-related changes in skull anatomy and conductivity may alter the spatial filtering of neural activity recorded at the scalp. Because ERPs, oscillatory activity, and aperiodic activity may arise from sources with different spatial distributions, these developmental changes could differentially influence their measured contributions to post-stimulus spectra. Studies spanning broader age ranges are therefore needed to establish whether the relationships observed here remain stable across development.

### Conclusion

Taken together, these findings demonstrate that stimulus-related aperiodic changes are a pervasive and consequential feature of the EEG signal that conventional time–frequency analyses are ill-equipped to handle. By assuming a stable aperiodic background across the peri-stimulus window, conventional approaches attribute broadband shifts to frequency-specific oscillatory dynamics, systematically overestimating theta and mischaracterizing beta and gamma. Alpha desynchronization is the exception, a robust periodic phenomenon that survives and is enhanced by correction, providing internal validation that correction does not simply abolish all spectral effects. These findings do not imply that theta-, beta-, or gamma-band activity has no oscillatory contribution; rather, they show that band-limited power changes alone cannot establish the presence of a separable oscillation and call for explicit characterization of narrowband and broadband spectral components. As methods for aperiodic parameterization mature, their routine use in event-related paradigms will be essential for an accurate understanding of the neural dynamics underlying cognition and behavior.

## Data Availability

The dataset supporting the conclusions of this article is available in the “SFARI_EEG_multi-paradigm dataset” repository (BIDS format), doi:10.18112/openneuro.ds006780.v1.0.0. The scripts used for preprocessing and analyses of the data are available on GitHub: https://github.com/tvanneau/Aperiodic_Theta
